# Delayed Replantation of Avulsed Teeth: Two Case Reports

**DOI:** 10.1155/2015/197202

**Published:** 2015-02-25

**Authors:** Selcuk Savas, Ebru Kucukyilmaz, Merve Akcay, Serhat Koseoglu

**Affiliations:** ^1^Department of Pediatric Dentistry, Faculty of Dentistry, Izmir Katip Celebi University, Cigli, 35640 Izmir, Turkey; ^2^Department of Periodontics, Faculty of Dentistry, Izmir Katip Celebi University, Izmir, Turkey

## Abstract

This case report presents two cases of delayed replantation of avulsed maxillary central incisors after an extended dry extra-alveolar period. Eight-year-old boy and 10-year-old boy presented with avulsed maxillary central incisors due to trauma occurring 27 and 7 hours earlier, respectively. Treatment guidelines for avulsed mature/immature permanent teeth with prolonged extra-oral time were carried out for the teeth and the extra-oral endodontic treatment was completed. After having been repositioned, the teeth were stabilized for 4 weeks and prophylactic antibiotic was prescribed. Clinical and radiographic controls were done after 18 months for Case I and 12 months for Case II. During the follow-up periods the teeth reported in these cases have remained in a stable, functional position but revealed clinical initial replacement resorption and ankylosis.

## 1. Introduction

Tooth avulsion is complete displacement of a tooth from its socket and is seen in 0.5–3% of all dental injuries [1–3]. The prevalence of avulsion cases in children increases between the ages of 7 and 9 years due to incomplete root development and minimal resistance of the alveolar bone/periodontal ligament (PDL) against extrusive forces during the eruption period of the teeth [1, 3].

The etiology of tooth avulsion varies according to the type of dentition. Avulsion in primary dentition is typically a result of hard objects hitting the teeth, whereas avulsion in permanent dentition is generally a result of falls, fights, sport injuries, automobile accidents, and child abuse [4–6].

In permanent and primary dentition, avulsion generally occurs in the maxilla, and the most commonly affected teeth are the maxillary central incisors. Increased overjet and incompetent lips were identified as potential etiological factors in such avulsion cases [2, 4, 7]. Although avulsion usually involves a single tooth, tooth-supporting tissue injuries, lip injuries, and multiple avulsions have also been documented [8, 9].

The primary goal in treating an avulsed tooth is to preserve and treat the supporting tooth tissues and to replant the avulsed teeth. The success of replantation depends on the patient's general health, the maturity of the root, the time the tooth is out of its socket, and storage medium [10–13]. The period of extra-oral time and the storage medium have the most critical effect on the status of the PDL cells [11–13]. The aim of this case report was to present two cases of delayed replantation of avulsed maxillary central incisors after an extended dry extra-alveolar period.

## 2. Case Reports

### 2.1. Case I

**Figure 1:**
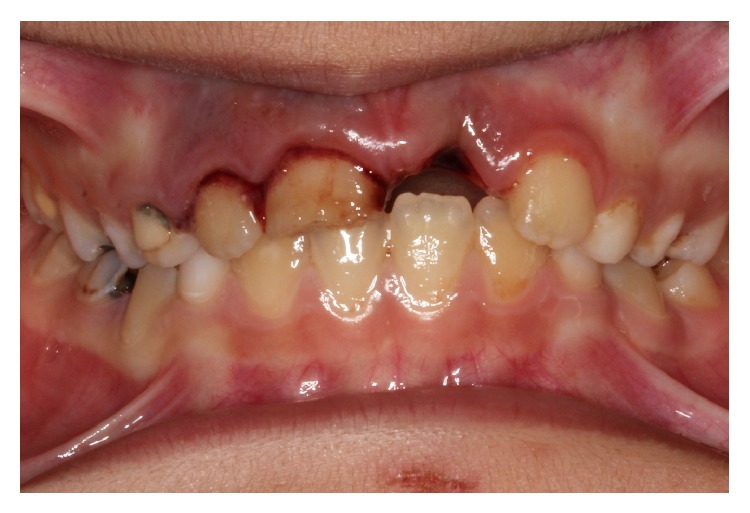
Avulsion of the left upper incisor.

An 8-year-old boy was referred to the pediatric dental clinic after a fall that resulted in dental trauma. The trauma occurred 27 hours ago while the child was playing in the school garden. The child had already been seen by the medical staff of the emergency unit of a local hospital who had detected no neurological damage or medical complications. His parents had let the avulsed tooth dry in a piece of paper and brought it to the clinic. Any concomitant systemic disease is not defined by the patient's parents. The intraoral examination revealed that the maxillary left permanent central incisor (tooth 21) was avulsed (Figure 1). An uncomplicated crown fracture, with dentin involvement, luxation, hypermobility of the upper right permanent central incisor (tooth 11), and laceration of the palatal mucosa, was detected. In a vitality test, the adjacent teeth gave a positive response. The patient had mixed dentition and severe carious lesions due to poor oral hygiene.

Periapical and panoramic radiographs revealed no alveolar bone wall fracture or other hard tissue injuries. Examination of the avulsed tooth showed that the crown had an enamel fracture, the root had an open apex, and the root surface was covered with dried remnants of periodontal tissue.

**Figure 2:**
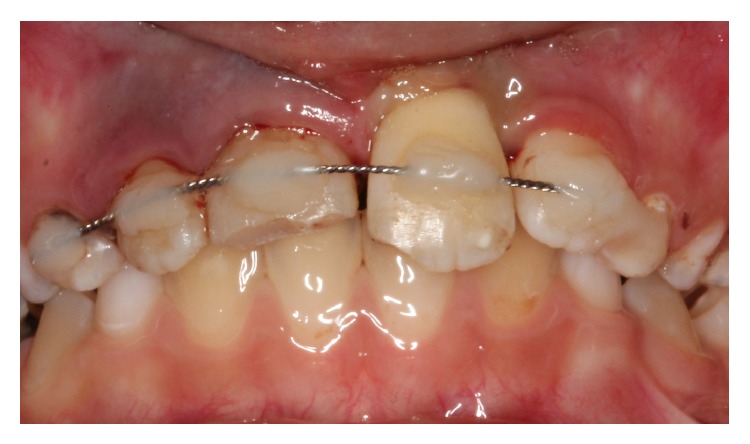
Splinting of the avulsed tooth with orthodontic wire and composite resin.

**Figure 3:**
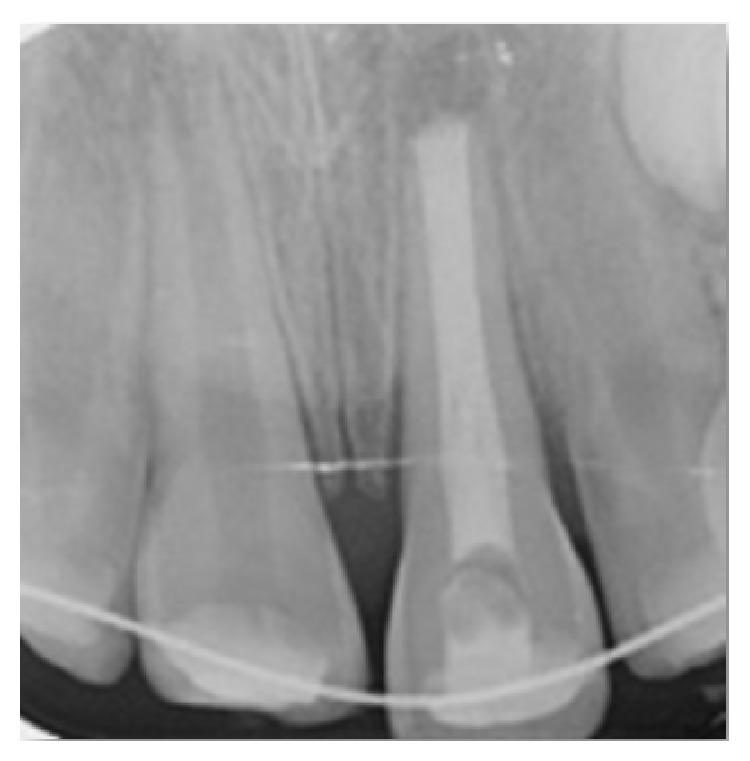
Periapical radiograph after immediate replantation of avulsed tooth.

**Figure 4:**
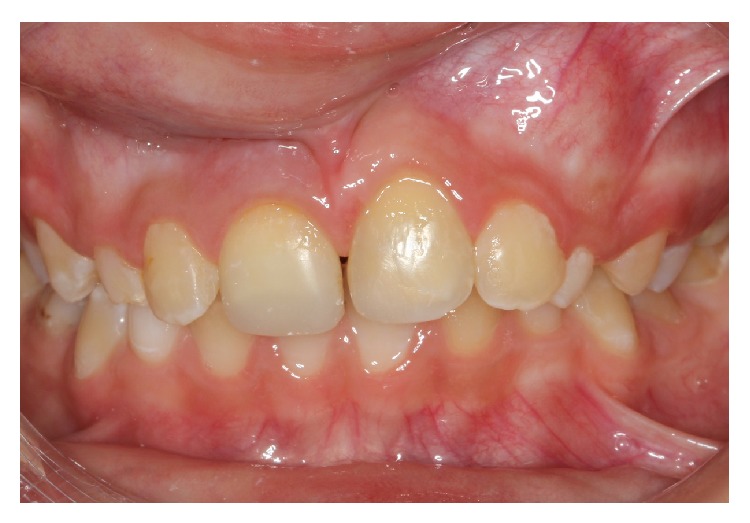
Frontal view, 18 months after trauma, slight infraposition of avulsed tooth.

**Figure 5:**
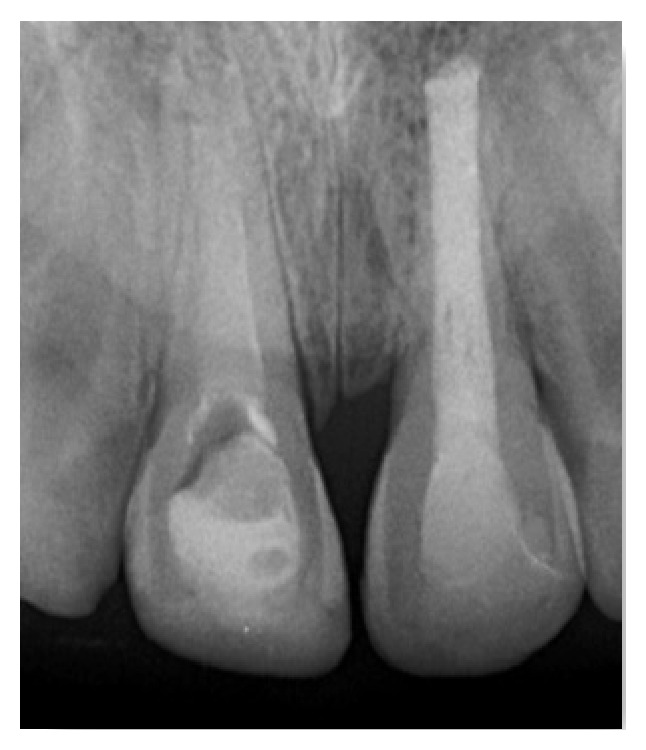
18-months follow-up of replanted tooth.

After informing the parents of the patient about possible risks, the socket of the tooth was gently rinsed with saline solution under local anesthesia (Maxicaine, Vem Drugs, Istanbul, Turkey). The root of the tooth was cleaned carefully to remove necrotic and dried remnants of periodontal tissue. Extra-oral endodontic treatment was carried out on the tooth, the root canals were filled with mineral trioxide aggregate (MTA) (BioAggregate, DiaDent, Burnaby, BC, Canada), and the tooth was replanted slowly, with slight digital pressure. Moist cotton pellet and glass ionomer cement (Ketac Molar, 3M/ESPE Dental Products, St. Paul, MN, USA) were used to restore the access cavity temporarily. The position of the replanted tooth was verified both clinically and radiographically. The tooth was stabilized using a flexible splint (0.195 inch round twist-flex arch wires, 3M Unitek, Monrovia, CA, USA) and the acid-etch composite resin technique (Clearfil Majesty Esthetic, Kuraray, Tokyo, Japan) (Figures 2 and 3). Moreover, oral hygiene instructions and advice about a soft diet and the need to use a chlorhexidine (Klorhex, Drogsan, Ankara, Turkey) mouth rinse during the stabilization period were provided at this time. Prophylactic antibiotic therapy with amoxicillin trihydrate/potassium clavulanate (Beecham Laboratories, Bristol, TN, USA) at a dose of 625 mg/day was prescribed for one week. The patient was also referred for an antitetanus booster. The parents were informed about the importance of regularly returning for clinical and radiographic follow-up. The patient was reviewed after two weeks, and no clinical or radiological pathological changes were detected. The patient was seen again four weeks after replantation, and the splinting wire was removed at this appointment. The permanent restoration of the fractured teeth crowns was completed with resin composite (Clearfil Majesty Esthetic, Kuraray Tokyo, Japan). In the third month follow-up, a percussion test of the avulsed tooth revealed a change in the percussion sound due to ankylosis. At a recall visit 12 months later, the right central incisor gave a negative response in a vitality test. An apexification procedure using calcium hydroxide (Sultan Chemists Inc., Englewood, NJ, USA) was started to induce apical closure. During an 18-month follow-up period, the replanted tooth remained in a stable, functional position but showed initial replacement resorption, ankylosis, and approximately 0.5 mm infraocclusion (Figures 4 and 5). The patient will be monitored till her growth is complete and appropriate treatment will be carried out if needed. CBCT images were taken to evaluate the relationship between the lateral incisor and permanent canine roots.

### 2.2. Case II

**Figure 6:**
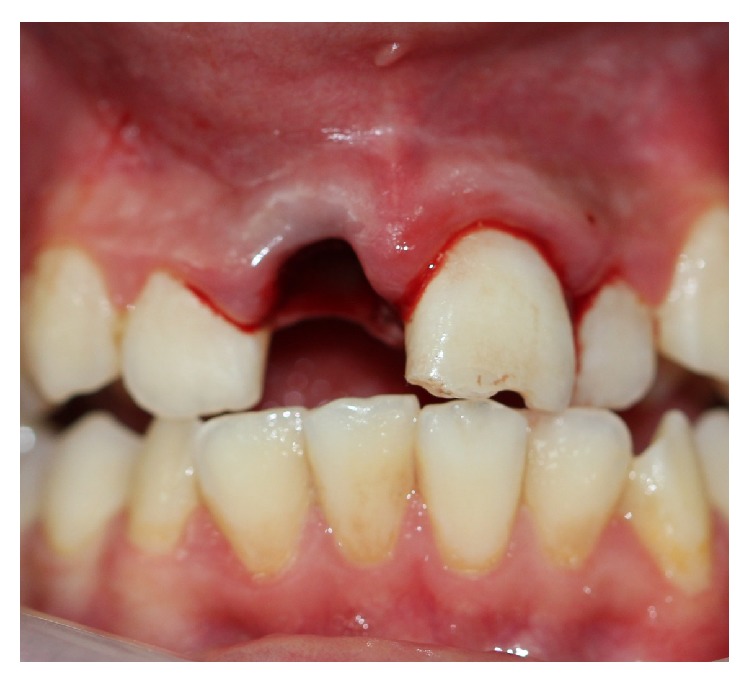
Avulsion of the right upper incisor.

A 10-year-old boy was referred to the pediatric dental clinic after a bicycle accident that resulted in dental trauma. The avulsed tooth was not placed in any storage medium and was brought to the clinic dry 7 hours after the accident. Any concomitant systemic disease is not defined by the patient's parents; there was no history of loss of consciousness or vomiting. On examination, no extra-oral injury was detected. The intraoral examination revealed that the maxillary right permanent central incisor (tooth 11) was avulsed (Figure 6). The left central incisor (tooth 21) showed enamel cracking and fracture. The patient had permanent dentition, with mild crowding and incisal overjet. No carious lesions were detected clinically, and his oral hygiene was fair.

Periapical and panoramic radiographs revealed no alveolar bone fracture. Examination of the avulsed tooth revealed that the crown had an enamel fracture and that the root had a closed apex.

**Figure 7:**
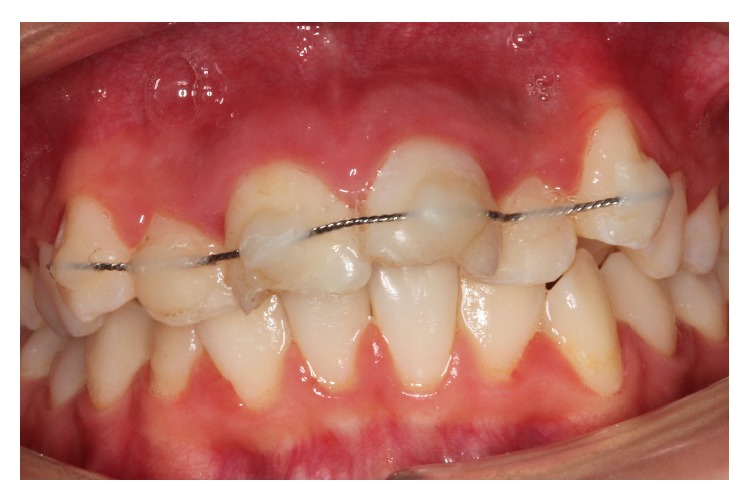
Splinting of the avulsed tooth with orthodontic wire and composite resin.

**Figure 8:**
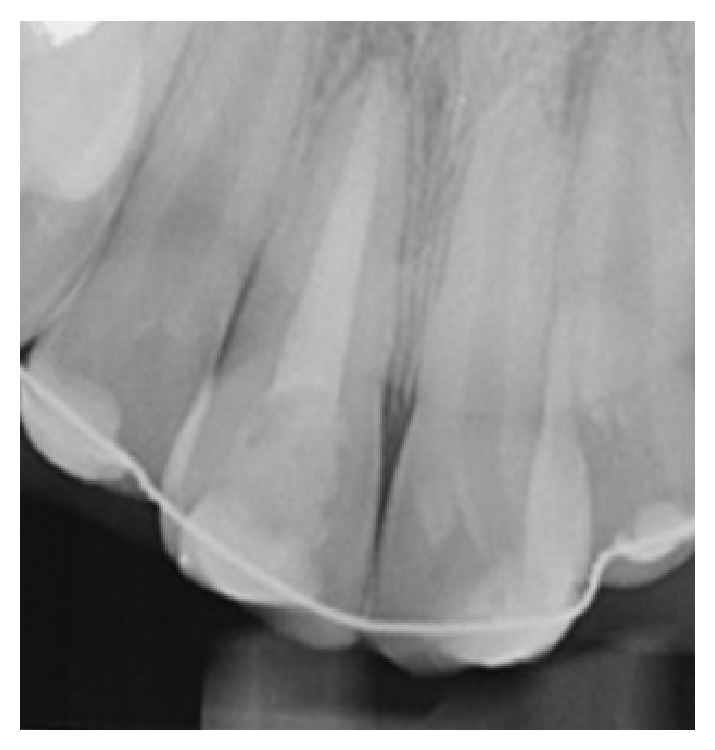
Periapical radiograph after replantation of avulsed tooth.

After examination, the treatment guideline for avulsed permanent teeth with closed apexes and prolonged extra-oral time for avulsed teeth were followed [3]. The root canal treatment was completed at this appointment extra-orally, and the root filling was done with MTA. A moist cotton pellet and glass ionomer cement (Ketac Molar, 3M/ESPE Dental Products, St. Paul, MN, USA) were used to restore the access cavity temporarily. The necrotic and dried remnants of periodontal tissue were carefully removed from the root surface of the tooth. Local anesthetic was administered, and the empty socket was thoroughly irrigated with sterile physiological saline. After removing coagulum from the socket, the tooth was replanted using light pressure. A periapical radiograph was taken to ensure that the tooth had been correctly positioned in the socket (Figure 7). The teeth were splinted from canine to canine with a flexible splint (0.195 inch round twist-flex arch wires) (Figure 8). The instructions given to the patient's family were as described in Case I (diet suggestions and oral hygiene instructions). In addition, prophylactic antibiotic therapy with amoxicillin trihydrate/potassium clavulanate at a dose of 1000 mg/day was prescribed for one week. The parents were informed about the importance of maintaining meticulous oral hygiene and regularly returning for clinical and radiographic follow-up.

**Figure 9:**
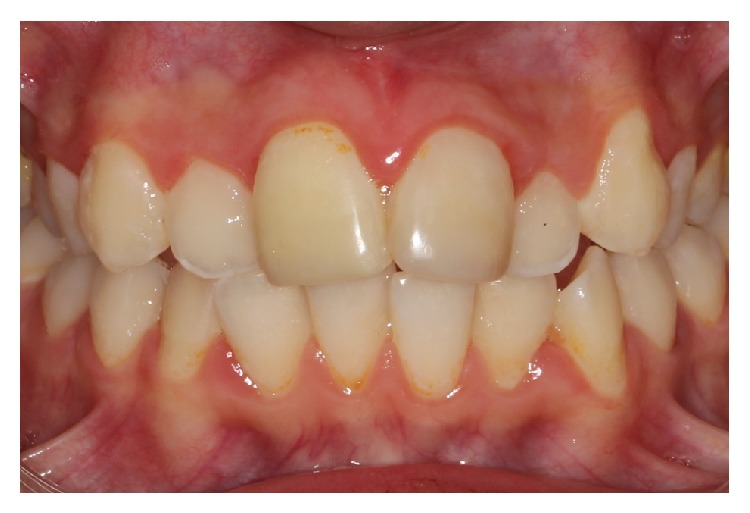
Frontal view, 12 months after trauma.

**Figure 10:**
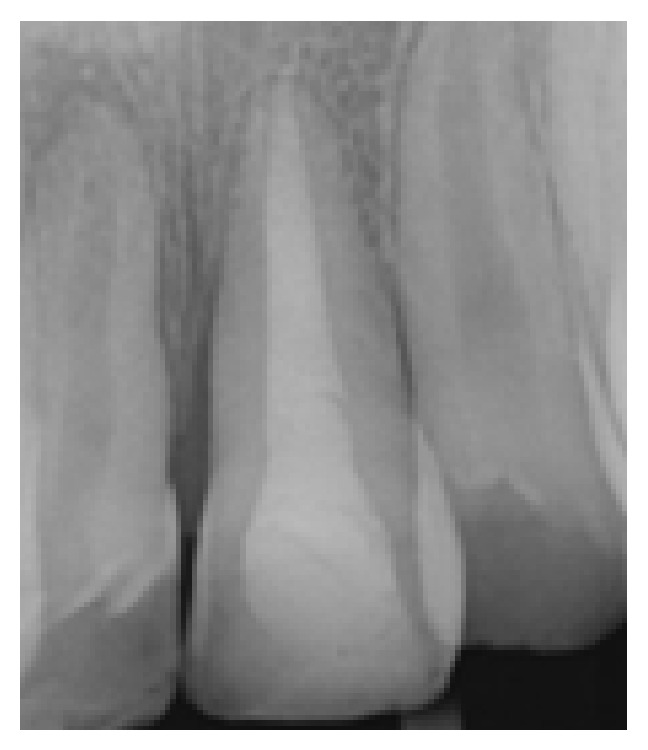
No pathology and resorption, 12-month radiographic examination.

Two weeks after replantation, the patient was reviewed, and no clinical or radiological evidence of pathological changes was detected. The patient was seen again four weeks after replantation, and the splinting wire was removed at this appointment. The permanent restoration of the fractured teeth crowns were completed with composite resin. At a recall visit three months later, ankylosis of the replanted tooth was observed with a percussion test. Clinical and radiographic controls were performed at six and 12 months. During the 12-month follow-up, clinical and radiographic examinations showed satisfactory functional and esthetic values for the avulsed tooth but some initial resorption and ankylosis with no infraocclusion (Figures 9 and 10). The patient will be monitored till her growth is complete and appropriate treatment will be carried out if needed.

## 3. Discussion

The guidelines for the treatment of avulsed permanent teeth vary, but the consensus is that the ideal treatment for an avulsed tooth is immediate replantation [3, 4]. However, it cannot always be carried out immediately.

The treatment decision regarding avulsed teeth is related to the maturity of the root apex (open or closed) and the condition of the PDL cells. The condition of PDL cells depends on the storage medium and the time the tooth has been out of the mouth [10, 11, 14–16]. The extra-oral period significantly affects the outcome and has a direct correlation with the survival of PDL cells. Clinical studies have indicated that teeth replanted within 5 minutes after avulsion have the best prognosis [17]. After a dry time of 60 minutes or more, all PDL cells are nonviable [3, 4]. The storage and transport media during the extra-oral time are also of vital significance. In patients with a prolonged extra-oral time, the tooth should be maintained in a suitable media, such as HBSS, saline, milk, or saliva until it is replanted by a dentist [18, 19].

In the present cases, the teeth were kept in dry pieces of paper, and the extra-oral dry time was more than 60 minutes (27 hours and 7 hours in Cases I and II, resp.). The management of the two cases presented here was in accordance with the accepted replantation protocol described by the International Association of Dental Traumatology [3]. It is indicated that, if the tooth has been dry for more than 60 min before replantation, the root canal treatment may be done extra-orally prior to replantation or later. Because there were no chances of obtaining pulp space revascularization and the periodontal ligament will be necrotic and not expected to heal, it was decided to treat the root canals extra-orally.

According to traumatology guidelines and articles on delayed replantation cases, PDL cells will be necrotic following delayed replantation, resulting in a poor long-term prognosis [1, 3, 4, 20]. Most avulsion trauma occurs before the patient's facial growth is completed. Preventing resorption of the surrounding bone and maintaining the tooth in the space of the arch are critical until facial growth is completed. Replantation can restore the patient's esthetic appearance and occlusal function and prevent physiological trauma, which may be associated with a missing anterior tooth. If the avulsed incisors had not been replanted in the present cases, other treatment options might have included prosthetic replacement of the missing incisor, space closure with orthodontic treatment, or autotransplantation of another tooth to the empty space.

Replanted teeth must be monitored carefully and clinical/radiographical findings should be recorded. In children and adolescents, ankylosis is frequently associated with infraposition of the replanted tooth. The replanted teeth of both cases presented here showed signs of ankylosis. Although Case II did not show infraposition, slight infraposition was visible in Case I compared with the adjacent central incisor. Decoronation may be necessary later when the degree of infraposition increases more than 1 mm.

## 4. Conclusion

Despite an extended extra-alveolar dry storage time, teeth with delayed replantation might be retained in a stable and functional position in the dental arch. In patients for whom growth has not ceased, using the replanted tooth to maintain the surrounding bone for a few years until the patient is a viable implant candidate can be considered a suitable therapeutic option.
